# *Bacillus megaterium* Renuspore^®^ as a potential probiotic for gut health and detoxification of unwanted dietary contaminants

**DOI:** 10.3389/fmicb.2023.1125616

**Published:** 2023-04-11

**Authors:** Annie Simon, Joan Colom, Shahneela Mazhar, Ekaterina Khokhlova, John Deaton, Kieran Rea

**Affiliations:** ^1^ADM Cork H&W Ltd., Bioinnovation Unit, University College Cork, Cork, Ireland; ^2^Deerland Probiotics and Enzymes/ADM, Kennesaw, GA, United States

**Keywords:** probiotic, *Bacillus*, megaterium, gut, microbiota

## Abstract

Exposure to diverse environmental pollutants and food contaminants is ever-increasing. The risks related to the bioaccumulation of such xenobiotics in the air and food chain have exerted negative effects on human health, such as inflammation, oxidative stress, DNA damage, gastrointestinal disorders, and chronic diseases. The use of probiotics is considered an economical and versatile tool for the detoxification of hazardous chemicals that are persistent in the environment and food chain, potentially for scavenging unwanted xenobiotics in the gut. In this study, *Bacillus megaterium* MIT411 (Renuspore^®^) was characterized for general probiotic properties including antimicrobial activity, dietary metabolism, and antioxidant activity, and for the capacity to detoxify several environmental contaminants that can be found in the food chain. *In silico* studies revealed genes associated with carbohydrate, protein and lipid metabolism, xenobiotic chelation or degradation, and antioxidant properties. *Bacillus megaterium* MIT411 (Renuspore^®^) demonstrated high levels of total antioxidant activities, in addition to antimicrobial activity against *Escherichia coli, Salmonella enterica, Staphylococcus aureus*, and *Campylobacter jejuni in vitro*. The metabolic analysis demonstrated strong enzymatic activity with a high release of amino acids and beneficial short-chain fatty acids (SCFAs). Moreover, Renuspore^®^ effectively chelated the heavy metals, mercury and lead, without negatively impacting the beneficial minerals, iron, magnesium, or calcium, and degraded the environmental contaminants, nitrite, ammonia, and 4-Chloro-2-nitrophenol. These findings suggest that Renuspore^®^ may play a beneficial role in supporting gut health metabolism and eliminating unwanted dietary contaminants.

## 1. Introduction

While the rapid industrialization and urbanization of the last century have improved many facets of everyday life, there has been negative consequent contamination of soil and water resources which have impacted the food chain and human health (Zhao et al., 2005). Several pollutants that are highly toxic and difficult to degrade including heavy metals and xenobiotics such as flavorings, food additives, pesticides, and industrial chemicals are detectable in water and food (Atashgahi et al., 2018; Feng et al., 2018; Srednicka et al., 2021). These environmental contaminants can negatively impact human and animal physiology affecting various organs and tissues and exacerbating undesirable symptoms including inflammation (Madani et al., 2015), oxidative stress (Nuran Ercal et al., 2005), and intestinal problems (Breton et al., 2013). As such, exclusion, degradation, or removal of these products from the food chain is of ever-increasing interest.

Several remedies have been developed to alleviate this phenomenon including the use of probiotics to protect against foodborne environmental contaminants (Feng et al., 2018). Probiotics are live microorganisms that benefit human health when supplemented in adequate amounts (Quigley, 2010). Probiotics possess health-promoting properties including antimicrobial activity, antioxidant and anti-inflammatory properties, immunomodulatory capacity, and production of bioactive molecules such as enzymes, short-chain fatty acids, and free amino acids among others (Minj et al., 2021). In addition, probiotic bacteria may possess intrinsic genetic machinery for eliminating environmental contaminants from the human body (Bisanz et al., 2014). Clinical research performed on pregnant women and children in Tanzania has suggested that probiotic yogurt can protect mothers and children from heavy metal poisoning (Bisanz et al., 2014). A review on the remediation of gut contaminants has also shown that *Lactobacilli* can alleviate toxicity caused by cadmium and decrease oxidative stress in cells (Feng et al., 2018). Among *Lactobacillus* species, *L. rhamnosus* GG is a widely researched probiotic strain that possesses most of the probiotic attributes, such as acid and bile resistance, adhesion ability, antimicrobial activity, antioxidant capacity, toxin removal ability, and heavy metal biosorption capability (Halttunen et al., 2008). *L. rhamnosus* GG has also been studied in the gut remediation of parathion pesticide and the degradation of aflatoxin from fungus (Feng et al., 2018; Westerik et al., 2018). Similarly, different spore probiotic species such as *B. subtilis, B. megaterium, B. coagulans*, and *B. pumilus* were also investigated for the removal of environmental contaminants using chelation or bioaccumulation principle (Elshaghabee et al., 2017). Interestingly, *Bacillus megaterium* has been studied for the elimination of environmental pollutants because of high levels of resistance to hostile conditions including exposure to heavy metals (Norma, 2018), with some strains demonstrating efficacy in the degradation of bisphenol A (BPA) present in industrial effluents, soil, and sludge (Suyamud et al., 2018).

In this study, we evaluated the probiotic properties of *B. megaterium* MIT411 (Renuspore^®^) and its impact on various aspects of host physiology, and the mechanisms involved in the bioremediation of environmental contaminants. For comparison, we used *L. rhamnosus* GG (ATCC 53103) or *L. fermentum* (obtained from a commercial product) as controls for our *in vitro* studies. We first interrogated the genomics of Renuspore^®^ to identify specific genes, pathways, and enzymes that are responsible for the bioaccumulation or degradation of environmental contaminants and addressed their functional activity in the bioaccumulation of heavy metals and degradation of xenobiotics and finally investigated their mechanisms in bioremediation. In addition, *in silico* analysis of the enzymes involved in the proteolytic, lipolytic, and carbohydrate metabolism pathways was performed, and a diverse enzymatic profile which includes protease activity, production of short-chain fatty acids (SCFA), and free amino acids (FAA) was demonstrated. These *in silico* findings were then tested with *in vitro* assays for antimicrobial, anti-inflammatory, antioxidant, and enzymatic capabilities to determine their potential as a therapeutic probiotic.

## 2. Materials and methods

### 2.1. Bacterial strains

Renuspore^®^, a spore-forming, Gram-positive, rod-shaped bacteria, was obtained from Deerland Probiotics and Enzymes/ADM, Kennesaw, USA. Renuspore^®^ was subjected to 16S DNA sequencing by Eurofins genomics, Germany. NCBI GenBank and BLAST tools were used to confirm the sequences and species. Indicator strains, namely *Escherichia coli* 25922, *Salmonella enteritidis* 13076, *Pseudomonas aeruginosa* DSM3227, *Staphylococcus aureus* RF122, and *Campylobacter jejuni* DSM4688, were sourced from Deutsche Sammlung von Mikroorganismen und Zellkulturen (DSMZ), Germany. Comparator probiotic strain such as *Lactobacillus rhamnosus* GG was purchased from an American type cell culture collection (ATCC 53103), while *Lactobacillus fermentum* was isolated from a commercially available product. All strains, except for *L. fermentum*, were grown aerobically in tryptic soy broth (TSB) medium at 37°C for 18 h at 170 RPM. *L. fermentum* was grown under the same conditions using MRS broth. For counts, all strains were plated on tryptic soy agar (TSA) medium. The vegetative form of Renuspore^®^ (1 x 10^8^ CFU/ml) was used in all *in vitro* studies. In addition, spores of Renuspore^®^ (1 x 10^8^ CFU/ml) were used in the adhesion ability assay. All media were purchased from Sigma-Aldrich, Ireland Ltd.

### 2.2. Genomic analysis

The genes encoding antioxidants, heavy metals, nitrite degradation, and proteolytic, lipolytic, and carbohydrate metabolism pathways were analyzed from the genome sequence data, which were retrieved from NCBI GenBank (NZ_JABBNK010000001). The complete GenBank file was used in RAST subsystem technology to find and annotate protein-encoding genes (Jyoti et al., 2022). All the genes involved in protein and lipid metabolism were screened to facilitate comparison with metabolomic and enzymatic data. Each gene was cross-referenced with the Renuspore^®^ genome file for confirmation.

### 2.3. Antimicrobial activity

The antimicrobial activity of Renuspore^®^ (*B. megaterium* MIT411) was tested using the agar diffusion method (Balouiri et al., 2016). A measure of 10 μL of cultured Renuspore^®^ at 1 x 10^8^ CFU/mL concentration was inoculated at the center of the TSA agar plate, air-dried, and incubated at 37°C for 18 h. Post-incubation, the Renuspore^®^ spotted agar plates were overlayed with 5 mL of fresh molten TSA agar (0.4%) inoculated with 100-fold dilutions of cultured pathogen strains. The plates were incubated at 37°C for 24 h, and the zones of inhibition were recorded in mm. Control strains were not indicated in the assay as the antimicrobial activity was performed to establish probiotic property of Renuspore^®^.

### 2.4. Antioxidant and reduced glutathione activity

The total antioxidant capacity and reduced glutathione (GSH) activity of Renuspore^®^ and *L. rhamnosus* GG were quantified by colorimetric analysis at 570 nm and 420 nm, respectively, using the total antioxidant capacity (TAC) (MAK187, Sigma-Aldrich, Ireland) (Ibrahim et al., 2021) and Reduced Glutathione Assay Kits (E-BC-K030-S, Elabscience , China). For these studies, both strains were grown in the TSB medium at 37°C until a final concentration of 1x10^8^ CFU/mL was reached. The culture was then centrifuged at 6,000 RPM for 15 min, and the supernatant was discarded. The cell pellet was washed and resuspended in 1X PBS three times. The washed cell pellet was resuspended in 1.5 mL 1x PBS and homogenized three times at 3,500 RPM for 30 s using a microtube homogenizer (Beadbug, Benchmark, Ireland). The homogenized sample was centrifuged at 6,000 RPM for 15 min at 4°C. The cell debris was discarded, and the cell lysate was recovered for testing. 1X PBS was used as a negative control. The TAC and reduced glutathione assay were performed following the manufacturer's instructions.

### 2.5. Adhesion study

HT-29 (a human colorectal adenocarcinoma cell line) and mucous-secreting cell line HT-29-MTX (ATCC), purchased from ATCC, were routinely maintained in DMEM low glucose medium supplemented with 10% fetal bovine serum, 1% L-glutamine, and antimicrobials such as penicillin (100 U/mL) and streptomycin (100 μg/mL). Cell lines were incubated in a 5% CO_2_ atmosphere at 37°C. Cells were seeded onto 24-well plates at a density of 5 x 10^5^ cell/well and cultured for 21–28 days to complete maturation.

Before the experiment, cells were washed two times with 0.5 mL DPBS, and 0.4 mL of full media without antibiotics was added to each well. Bacterial cells were added to the cell lines, and the adhesion ability was determined by bacterial cell counts in CFU/mL.

Since the adhesion ability of *L. fermentum* has been well-established and also has two times higher adhesion capacity to *L. rhamnosus* GG in HT-29 cell lines (Gharbi et al., 2019), bacterial adhesion capacity to HT-29 and HT-29 MTX cell lines was investigated in Renuspore^®^ and *L. fermentum*. Renuspore^®^ (vegetative and spores) and *L. fermentum* with a final concentration of 10^8^ CFU/mL and 10^7^ CFU/mL, respectively, were added to the cell lines. The bacterial suspension and cell lines were incubated for 3.5 h at 37°C in a humidified atmosphere at 5% CO_2_. Controls were prepared in a similar way without the cell lines. Bacterial adherence ability was assessed at 37°C. After incubation, the cell layers were washed four times with DPBS to remove non-adherent bacteria, before the cell layers were detached by the addition of Trypsin/EDTA (Sigma-Aldrich, USA) (Letourneau et al., 2011; Gagnon et al., 2013; Tsilia et al., 2015). The remaining suspensions with viable adhered bacteria were diluted and plated onto TSA or MRS agar. The number of colony-forming units was counted after aerobic incubation at 37°C for 24 h.

### 2.6. Heavy metal bioaccumulation

#### 2.6.1. Mercury and lead

Renuspore^®^ and *L. rhamnosus* GG were cultured aerobically in TSB medium (Sigma-Aldrich, Ireland Ltd.) supplemented with 1 ppm of lead or mercury (Sigma-Aldrich, Ireland Ltd.) until they reached a final concentration of 1 × 10^8^ CFU/mL. Cultures were centrifuged at 6,000 RPM for 15 min at room temperature, and the supernatants were saved. TSB medium supplemented with 1 ppm lead or mercury was set as control. Saved supernatants and controls were filter-sterilized and sent to Eurofins Food Testing Ireland Limited for quantification by ICP-MS analysis. ICP-MS (Agilent 7900) consists of plasma, a mass spectrometer with a quadrupole mass filter, and a detector. All liquids were converted to aerosol by pumping into the nebulizer. The saved supernatants and control samples were carried in a gas stream of plasma in the form of an aerosol. The high-temperature electrical discharge of plasma transformed samples into vapors to atomize and ionize the elements. The ionized particles were then separated, and the analyte ions were filtered by the quadrupole mass filter. The filtered mass was finally counted by the detector (González-Antuña et al., 2017).

#### 2.6.2. Iron, calcium, and magnesium bioaccumulation

Renuspore^®^ and *L. rhamnosus* GG were cultured aerobically in TSB medium (Sigma-Aldrich, Ireland Ltd.) supplemented with 500 μM of calcium and 10 ppm of iron or magnesium (Sigma-Aldrich, Ireland Ltd.), until they reached a final concentration of 10^8^ CFU/mL. Cultures were centrifuged at 6,000 RPM for 15 min at room temperature, and the supernatants were saved. TSB medium supplemented with 500 μM of calcium and 10 ppm of iron or magnesium was set as control. Saved supernatants and controls were filter-sterilized and analyzed using an Iron Assay Kit (Sigma-Aldrich, MAK025, Ireland Ltd.), Calcium Assay Kit (Cell Biolabs, MET5121, USA), and Magnesium Microplate Assay Kit (Cohesion biosciences, CAK1107), according to the manufacturer's instructions.

### 2.7. Xenobiotic degradation

#### 2.7.1. Nitrite detoxification

Renuspore^®^ and *L. rhamnosus* GG were cultured in TSB supplemented with 100 μM nitrite solution (Sigma-Aldrich, 72586, Ireland Ltd.) until they reached 10^8^ CFU/mL. Cultures were centrifuged at 6,000 RPM for 15 min at room temperature, and the supernatants were saved. TSB medium with 100 μM nitrite solution was set as control. The saved supernatants and control were filter-sterilized and analyzed using a Nitrite/Nitrate Assay Kit (Sigma-Aldrich, 23479, Ireland Ltd.). The levels of nitrite were measured at 570 nm, following the manufacturer's instructions.

#### 2.7.2. Ammonia degradation

To evaluate the growth and ability of Renuspore^®^ to metabolize ammonia, Renuspore^®^ and *L. rhamnosus* GG were inoculated in supplemented minimal media which contained ammonium chloride. Minimal media were supplemented with 2% of 1M glucose, 0.2% of 1M magnesium sulfate, and 0.01% of 1 M calcium chloride. The inoculated media were incubated at 37°C for 24 h at 170 RPM. The supplemented minimal media without any strain were set as control. Ammonia or ammonium present in the filter-sterilized control and supernatants of Renuspore^®^ and *L. rhamnosus* GG were measured at 620 nm using an Ammonia/Ammonium Assay Kit (Abbexa, abx298905, UK) following the manufacturer's instructions.

#### 2.7.3. 4-Chloro-2-nitrophenol (4C2NP) degradation

Renuspore^®^ (1 x 10^8^ CFU/mL) was cultured for 24 h in minimal media containing 10 mM glucose, 0.20 % of 1M magnesium sulfate, 0.01% of 1M calcium chloride, and various concentrations of the xenobiotic 4-Chloro-2-nitrophenol (0.25, 0.5, 1.0, 1.5 and 2.0 mM) dissolved in ethyl alcohol. Every 2 h, 2 mL of samples were collected and centrifuged at 6,000 RPM for 15 min to observe 4C2NP decolorization in the supernatant. The 4C2NP decolorization was measured with the decrease in the optical density at 405 nm, and the percentage of decolorization was calculated as described by Arora (2012) using the following calculation:

% Decolorization = (Initial Absorbance – Absorbance after time t) × 100/Initial Absorbance.

Control strain was not indicated in the assay since *L. rhamnosus* GG was unable to grow in the presence of 4C2NP in the supplemented minimal media.

### 2.8. Carbohydrate fermentation

To evaluate carbohydrate metabolism in Renuspore^®^, API 50 CH, a standardized system, comprising 49 biochemical tests, was used. Several pure identical single colonies of Renuspore^®^ were picked and suspended in an ampule of API NaCl 0.85% medium (Biomérieux, Ref 70700) until turbidity was equivalent to 2 McFarland (Biomérieux, Ref 70900). The prepared suspension was transferred to an ampule of API 50 CHB/E medium (Biomérieux, Ref 50430). Two hundred microliters of the inoculated API 50 CHB/E medium were added into the strip wells and incubated for 48 h. A positive test corresponded to acidification revealed by the phenol red indicator contained in the medium changing to a yellow color. Control strain was not indicated in the assay since carbohydrate fermentation was performed to analyze the potential probiotic properties of Renuspore^®^.

### 2.9. Enzymatic activity

The enzymatic profiling was performed by API ZYM (API Laboratory Products Ltd., Biomerieux, France), a semi-quantitative method, comprising 19 naphthyl substrates to evaluate the enzymatic activities of Renuspore^®^. Several pure identical single colonies of Renuspore^®^ were picked and suspended in an ampule of API suspension medium (Biomérieux, Ref 70700) until turbidity was equivalent to 6 McFarland (Biomérieux, Ref 70900). Two hundred microliters of the inoculated API suspension medium were added into the strip wells and incubated for 48 h. Post-incubation, the reactions were read on the color chart according to the manufacturer's manual. Control strain was not indicated as the assay was performed to determine the enzymatic activity of the probiotic Renuspore^®^.

### 2.10. Protease activity

The quantitative extracellular protease activity in the solution was investigated in Renuspore^®^ by employing a commercial EnzChek^®^ Protease Assay Kit (Molecular Probes, Eugene, OR, USA), which contains a casein derivative labeled with green-fluorescent BODIPY^®^ FL dye.

The cells of Renuspore^®^ and *L. rhamnosus* GG (1 x 10^8^ CFU/mL) were washed and suspended in 100 μl of 1X PBS for the assay. Proteinase K was used as a positive control, and all samples were assayed with an incubation time of 24 h using the EnzChek^®^ Protease Assay Kit, according to the manufacturer's instructions. Fluorescence (Ex/ Em 485/ 535 nm) was measured on a Tecan Microplate Reader using standard filters.

### 2.11. Free amino acids (FAAs) and short-chain fatty acids (SCFAs)

Renuspore^®^ (1 × 10^8^ CFU/mL) was cultured in ultra-high-temperature (UHT) milk (Indomilk, Semarang, Central Java, Indonesia). The culture was incubated at 37 °C for 48 h with shaking. A cell-free supernatant was used to analyze FAA and SCFA. Control strain was not tested for FAA and SCFA since the objective of the assay was to investigate the metabolites of Renuspore^®^. The uninoculated UHT milk was used as a negative control. MS-Omics (Vedbæk, Denmark) performed mass spectrometry to analyze FAA and SCFA. Hydrochloride acid was used to acidify the sample, and deuterium-labeled internal standards were used for analysis. All samples were analyzed by GC (7890B, Agilent), combined with a quadrupole detector (5977B, Agilent) where a high polarity column (Zebron™ ZB-FFAP, GC Cap. Column 30 m × 0.25 mm × 0.25 μm) was installed. All raw data were formatted to net CDF *via* Chemstation (Agilent). The final data were imported and assessed in Matlab R2014b (Mathworks Inc.) through PARADISE software (Johnsen et al., 2017).

### 2.12. Statistical analysis

Degradation of the 4-Chloro-2-nitrophenol (4C2NP) data was analyzed using a two-way analysis of variance (ANOVA), followed by Dunnett's multiple comparison test. The free amino acid and short-chain fatty acid GC-MS results were analyzed using multiple *t*-tests using unpaired parametric, two-stage step— up (Benjamini, Krieger, and Yekutieli). All other results were statistically analyzed using the one-way analysis of variance (ANOVA) (biological replicates *n* = 3 or 5), followed by Tukey's multiple comparison test using GraphPad Prism v.9.

## 3. Results

### 3.1. Genomic analysis

Whole-genome sequencing analysis showed Renuspore^®^ genome is composed of 5,429,269 base pairs with no plasmids (using classic RAST annotations), and it encodes 5,429 coding genes and 171 RNA genes. Genes involved in the antioxidant activity (*Cu/Zn-SOD, Mn-SOD, Fe-SOD, Ni-SOD, CAT, GPx, PRDX, TXN, TXNRD*, and *NIR*) and heavy metal sequestration (*mer* and *ars*) have been associated with the *in vitro* total antioxidant capacity assay and ICP-MS heavy metal detection, respectively. In addition, genes linked to proteolytic activity and FAA and SCFA production were also observed. The number of possible genes encoding enzymes and transporters involved in carbohydrate metabolism and enzymatic activity was identified during genomic analysis. To correlate the genomic findings, the API 50 CH strips were employed for *in vitro* investigation. In carbohydrate fermentation, more than two genes were identified in the breakdown of D-ribose (*rbsA, rbsB, rbsD, rpiA*, and *rpiB)*, D-xylose (*xylA, xylE*, and *xylB)*, D-glucose (*Gdh, galT, galU, G6PD*, and GlcU), and D-trehalose (*treR, treB, thuA*, and *treA)*. Although Renuspore^®^ API 50CH results indicated positive fermentation of D-saccharose, D-maltose, D-melezitose, D-raffinose, amidon, glycogen, D-sorbitol, N-acetylglucosamine, and esculin ferric citrate (Table 1), the genes related to the metabolism of these carbohydrates were not identified in Renuspore^®^ genome (Table 1). Similarly, the API 50 CH identification kit was used to confirm the genomic findings of Renuspore^®^'s enzymatic profiling which included esterase activity (*EstA, fes, lpqC, paaI*, and *fadM*), lipase activity (*tesA*), peptidase activity (*oppA, oppF, DppB, DppD, DppC, pcp, Map, pepB, pepP, pepX, pepE, pepF, pepV*, and pepD), and glycosidase activity (*gala and galB*) (Table 2). Although the genomic analysis revealed genes for carbohydrate, protein, and lipid metabolism, the *in vitro* studies did not fully correlate with the genes identified.

**Table 1:** Renuspore^®^ can ferment 11 carbohydrates using API 50 CH strips, and the genes associated with the fermentation were indicated from the genomic analysis.

**Carbohydrates**	**–/+**	**Possible genes**	**Carbohydrates**	**–/+**	**Possible genes**
L-arabinose	+	*AraA, araD*	Salicin	–	
D-ribose	+	*rbsA, rbsB, rbsD, rpiA, rpiB*	D-cellobiose	–	
D-xylose	+	*xylA, xylE, xylB*	Inulin	–	
D-mannitol	+	*mtlD*	D-lactose	–	*Ldh, lutP*
D-glucose	+	*Gdh, galT, galU, G6PD*, GlcU	D-melibiose	–	
D-fructose	+	*pfkA, fsaA*	Glycerol	–	
D-trehalose	(+)	*treR, treB, thuA, treA*	Erythritol	–	
D-saccharose	+		D-arabinose	–	
D-maltose	+		Methyl-Ad-mannopyranoside	–	
D-melezitose	(+)		Xylitol	–	
D-rafinose	(+)		Gentiobiose	–	
Amidon	(+)		D-turanose	–	
Glycogen	(+)		D-Lyxose	–	
D-sorbitol	+		D-tagatose	–	
N-acetylglucosamine	+		D-fucose	–	
Esculin ferric citrate	+		L-fucose	–	
L-xylose	–		Methyl-Bd-xylopyranoside	–	
D-adonitol	–		D-galactose	–	*galB, galA*
L-sorbose	–		D-mannose	–	
L-rhamnose	–		Pottasium 5-ketogluconate	-	
Dulcitol	–		Arbutin	–	
Inositol	–	*idh*	D-arabitol	–	
Methyl-Ad-glucopyranoside	–		L-arabitol	–	
Pottasium 2-ketogluconate	–		Pottasium gluconate	–	
Amygdalin	–				

Legends indicate the following: +, positive; –, negative; (+), weakly positive.

**Table 2:** Semi-quantitative assay of enzyme activities of Renuspore^®^ using API ZYM kit (*n* = 3), where + indicates the presence and—indicates the absence of enzymes.

**Function**	**Possible genes**	**Enzymes**	**+/–**
Esterase activity	*EstA, fes, lpqC, paaI, fadM*	Esterase (C4:0)	**+**
		Esterase (C8:0)	**+**
Lipase activity	*tesA*	Lipase (C14:0)	**–**
Peptidase activity	*oppA, oppF, DppB, DppD, DppC, pcp, Map, pepB, pepP, pepX, pepE, pepF, pepV*, pepD	Leucine arylamidase	**–**
		Valine arylamidase	**–**
		Cystine arylamidase	**–**
Proteinase activity		Trypsin	**–**
		α-chymotrypsin	**+**
Phosphatase activity		Acid phosphatase	**–**
		Alkaline phosphatase	**+**
		Phosphohydrolyase	**+**
Glycosidase activity	*galA*	α-Galactosidase	**+**
	*galB*	β-Galactosidase	**+**
		β-Glucuronidase	**–**
		α-Glucosidase	**–**
		β-Glucosidase	**–**
		β-Glucosaminidase	**–**
		α-Mannosidase	**–**
		α-Fucosidase	**–**

### 3.2. Antimicrobial activity

A total of five opportunistic and zoonotic pathogens of the gut, the urinary tract, and the skin were selected for evaluating the antimicrobial properties of Renuspore^®^. Renuspore^®^ had an antimicrobial activity with a hazy zone of inhibition observed against *E. coli, S. enteritidis, S. aureus, and C. jejuni* on the TSA overlayed with 0.4% TSA agar (Table 3). No antimicrobial activity was observed against *P. aeruginosa*.

**Table 3:** Renuspore^®^ had moderate antimicrobial activity against *E. coli, S. enteritidis, S. aureus, and C. jejuni* in solid media (TSA).

**Renuspore^®^**	**Zone of inhibition (mm)**				
	***E. coli***	***S. enteritidis***	***S. aureus***	***C. jejuni***	***P. aeruginosa***
18.9 ± 0.9	18.7 ± 1.2	18.67 ± 0.58	21.1 ± 1.44	No inhibition

Antimicrobial activity is indicated as zone of inhibition (mm) ± standard deviation. The results were statistically analyzed by the Student's t-test analysis (biological replicates n =3) using GraphPad Prism v.9.

### 3.3. Antioxidant and reduced glutathione activity

**Figure 1:**
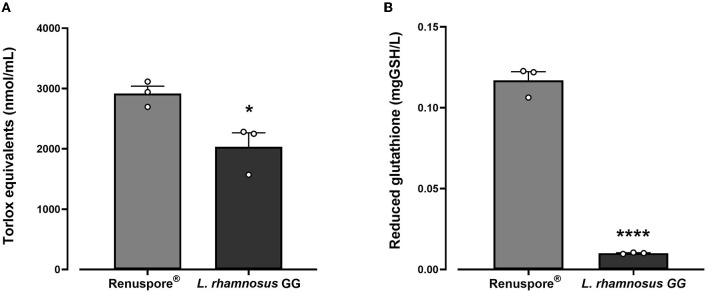
Renuspore^®^ had significantly increased levels of **(A)** Trolox equivalents and **(B)** reduced glutathione GSH compared with *L. rhamnosus* GG. The results show average concentration (*n* = 3) ± standard error. Significant difference observed between Renuspore^®^ and *L. rhamnosus* GG: *p < 0.05 and ****p < 0.0001. The results were statistically analyzed using one-way analysis of variance (ANOVA) (biological replicates *n* = 3) followed by Tukey's multiple comparison test using GraphPad Prism v.9.

From the genomic analysis, genes encoding antioxidant enzymes were identified in Renuspore^®^. Antioxidant genes such as superoxide dismutase (Cu/Zn-SOD, Mn-SOD, Fe-SOD, and Ni-SOD), catalase (CAT), glutathione peroxidase (GPx), peroxiredoxin (PRDX), thioredoxin (TXN), thioredoxin reductase (TXNRD), and nitrite reductase (NIR) were detected in Renuspore^®^. The genomic data were correlated with the *in vitro* models compared with *L. rhamnosus* GG. In *in vitro* antioxidant capacity and reduced glutathione activity assays, Renuspore^®^ (1 × 10^8^ CFU/mL) demonstrated superior antioxidant capacity [Figure 1, *F*_(2, 6)_ = 98.13, *p* < 0.0001] and increased mgGSH [Figure 1, *F*_(2, 6)_ = 443.7, *p* < 0.0001] output as compared with a known antioxidant probiotic *L. rhamnosus* GG.

### 3.4. Adhesion study

**Figure 2:**
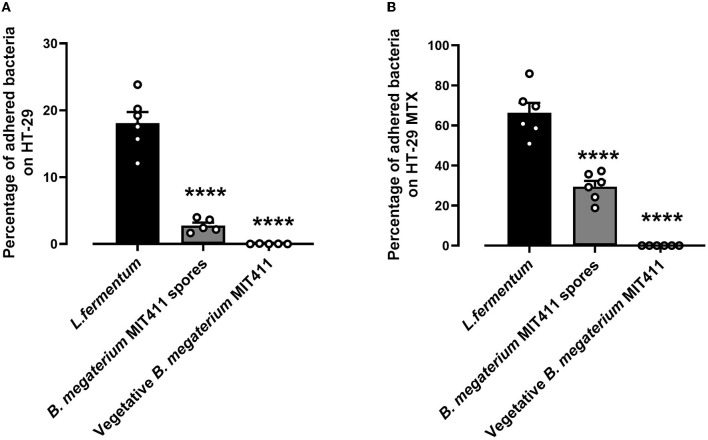
Renuspore^®^ does not significantly adhere to **(A)** HT-29 and **(B)** HT-29 MTX cells at 37°C compared with *L. fermentum*. Significant difference observed between Renuspore^®^ and *L. fermentum*. ****p < 0.0001. The results were statistically analyzed using Student's *t*-test analysis (biological replicates *n* = 6) using GraphPad Prism v.9.

The adhesion ability of Renuspore^®^ and *L. fermentum* (control) was studied in human colorectal adenocarcinoma cell lines HT-29 and HT-29-MTX at 37°C. Renuspore^®^ vegetative cells do not adhere to the HT-29 and HT-29 MTX at 37°C, whereas at 37°C, the spores of *B. megaterium* MIT411 adhered to the intestinal cell lines HT-29 [Figure 2A, *F*_(2, 13)_ = 81.60, *p* < 0.0001] and mucus-producing HT-29 MTX cell lines [Figure 2B, *F*_(2, 15)_ = 99.52, *p* < 0.0001]. *B. megaterium* MIT411 spores had low adhesion ability compared with *L. fermentum*.

### 3.5. Heavy metal bioaccumulation

**Figure 3:**
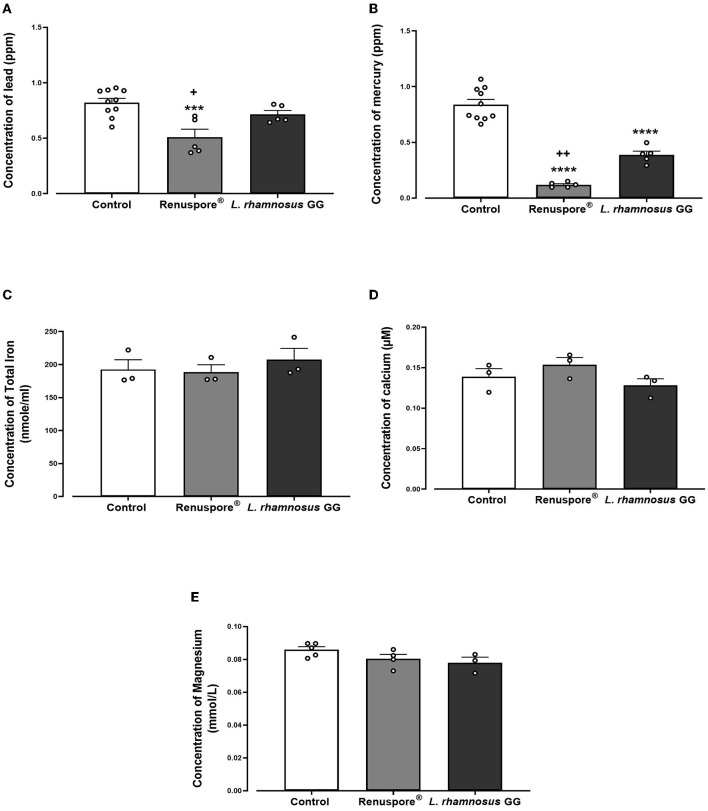
Renuspore^®^ significantly decreased the concentration of **(A)** lead and **(B)** mercury compared with *L. rhamnosus* GG. The results show average concentration (*n* = 5) ± standard error. Renuspore^®^ does not bioaccumulate **(C)** iron, **(D)** calcium, and **(E)** magnesium. The results show average concentration (*n* = 3) ± standard error. Significant difference observed between Renuspore^®^ and control is indicated: ***p < 0.001 and ****p < 0.0001. Significant reduction observed between Renuspore^®^ and *L. rhamnosus* GG is indicated: ^+^*p* < 0.05 and ^++^*p* < 0.01. The results were statistically analyzed using the one-way analysis of variance (ANOVA) (biological replicates *n* = 3 or 5) followed by Tukey's multiple comparison test using GraphPad Prism v.9.

When exposed to some of the most common toxic heavy metals in the environment, Renuspore^®^ effectively bioaccumulated lead [Figure 3A, *F*_(2, 17)_ = 10.71, *p* < 0.001] and mercury [Figure 3B, *F*_(2, 17)_ = 77.02, *p* < 0.0001] compared with control and probiotic strain *L. rhamnosus* GG. From Figure 3, in the presence of essential metals and minerals, Renuspore^®^ does not bioaccumulate iron [Figure 3C, *F*_(2, 6)_ = 0.4633, *p* > 0.5], calcium [Figure 3D, *F*_(2, 6)_ = 2.017, *p* < 0.5], and magnesium [Figure 3E, *F*_(2, 9)_ = 2.736, *p* < 0.5], thereby not compromising natural absorption of essential minerals in the body.

### 3.6. Xenobiotic degradation

#### 3.6.1. Nitrite detoxification

**Figure 4:**
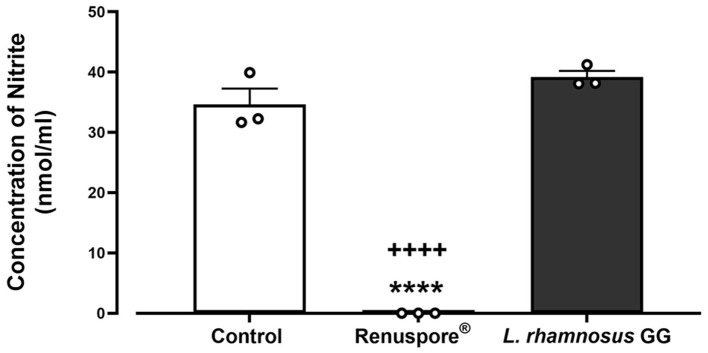
Renuspore^®^ reduced nitrite present in TSB medium compared with *L. rhamnosus* GG. Results show average concentration (*n* = 3) ± standard error. Significant reduction observed between control and Renuspore^®^ is indicated as ****p < 0.0001. Significant reduction observed between Renuspore^®^ and *L. rhamnosus* GG is indicated as ^++++^*p* < 0.0001. Results were statistically analyzed using the one-way analysis of variance (ANOVA) (biological replicates *n* = 3) followed by Tukey's multiple comparison test using GraphPad Prism v.9.

A genomic analysis of Renuspore^®^ reveals the presence of genes encoding the enzyme nitrite reductase (*NirD)*, which conventionally reduces nitrite to ammonium (Yukioka et al., 2017). In this study, *in vitro* analysis demonstrated the ability of Renuspore^®^ to reduce nitrite present in TSB medium below the limit of detection, whereas *L. rhamnosus* GG did not alter the concentration [Figure 4, *F*_(2, 6)_ = 168.9, *p* < 0.0001].

#### 3.6.2. Ammonia degradation

**Figure 5:**
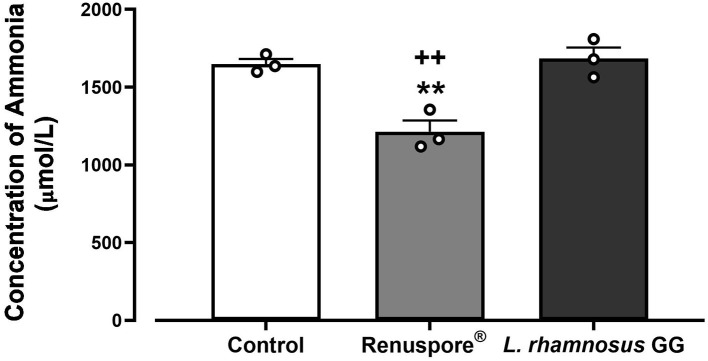
Renuspore^®^ degraded ammonia significantly in supplemented minimal media when compared with the untreated control and *L. rhamnosus* GG. The results show an average concentration (*n* = 3) ± standard error. The significance between Renuspore^®^ and control is indicated as **p < 0.01, and the significance between Renuspore^®^ and *L. rhamnosus* GG is indicated as ^++^*p* < 0.01. The results were statistically analyzed using the one-way analysis of variance (ANOVA) (biological replicates *n* = 3) followed by Tukey's multiple comparison test using GraphPad Prism v.9.

To test for the ability of Renuspore^®^ to degrade ammonia from the environment, Renuspore^®^ and the control strain *L. rhamnosus* GG were cultured in minimal media supplemented with ammonium chloride as the sole nitrogen source. Renuspore^®^ significantly decreased ammonia levels when compared with *L. rhamnosus* GG as determined by the Ammonia/Ammonium Assay Kit (Abbexa, UK) measured at 620 nm [Figure 5, *F*_(2, 6)_ = 18.10, *p* < 0.01].

#### 3.6.3. 4-Chloro-2-nitrophenol (4C2NP) degradation

**Figure 6:**
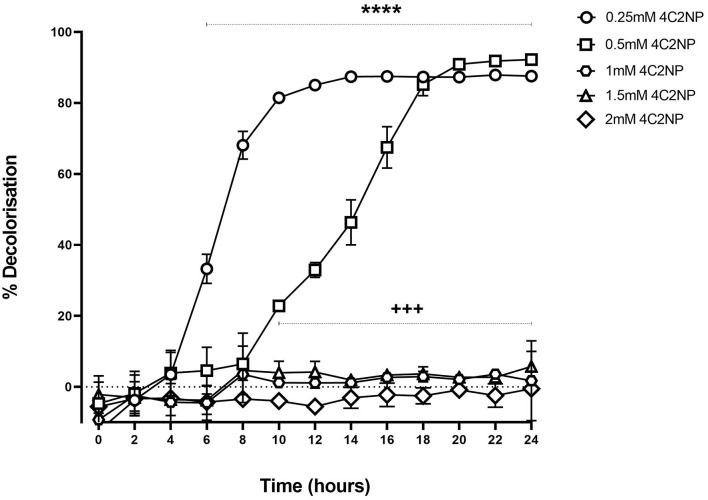
Renuspore^®^ degraded 4-Chloro-2-nitrophenol at various concentrations with respect to time by decolorization principal in minimal media supplemented with 10 mM glucose. The results show the average percentage of decolorization (*n* = 3) ± standard error. The significance between 6- and 24-h time points for the concentration at 0.25 mM 4C2NP is indicated as ****p < 0.0001. The significance between 10- and 24-h time points for the concentration at 0.5 mM 4C2NP is indicated as ^+++^*p* < 0.001. The results were statistically analyzed using the two-way analysis of variance (ANOVA) followed by Dunnett's multiple comparison test (biological replicates *n* = 3) using GraphPad Prism v.9.

Renuspore^®^ decolorized 4C2NP up to 0.5 mM concentration proving the degradation of the xenobiotic [Figure 6, *F*_(12, 52)_ = 19.96, *p* < 0.0001]. Renuspore^®^ rapidly decolorized at 0.25 mM concentration of 4C2NP in 10–16 h of incubation. At 0.5 mM concentration, Renuspore^®^ degraded 4C2NP in 18–24 h. Renuspore^®^ showed a limited decolorization at 1–1.5 mM concentration, and no decolorization at the concentration of 2.0 mM as Renuspore^®^ was unable to grow in these conditions (Figure 6).

### 3.7. Carbohydrate fermentation

Renuspore^®^ has the ability to ferment 11 carbohydrates out of the 49 tested carbohydrates using a commercial API 50 CH strip (Table 1). The *in vitro* analysis was confirmed with the genomic analysis and their possible genes encoding carbohydrate metabolism.

*In silico* analysis in the genome of Renuspore^®^ reported the presence of transporters and enzymes involved in the metabolism of D-ribose, L-arabinose, D-xylose, D-glucose, D-fructose, and D-mannitol, whereas enzymes for D-maltose, N-acetylglucosamine, D-saccharose, and D-sorbitol were not identified. In addition, genes involved in the metabolism of trehalose, lactose, galactose, inositol, and amylase A (*amyA*) involved in starch metabolism were also identified in the genome of Renuspore^®^ however, the results of the API test did not directly correlate with the presence of these genes.

### 3.8. Proteolytic activity

**Figure 7:**
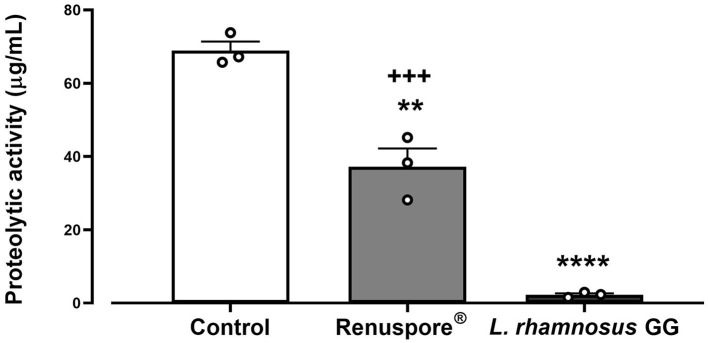
Renuspore^®^ demonstrates superior protease activity (μg/mL) to *L. rhamnosus* GG. The results show average concentration (*n* = 3) ± standard error. Significant difference observed between Renuspore^®^ and positive control (Proteinase K): **p < 0.01. Significant difference observed between Renuspore^®^ and *L. rhamnosus* GG: ^+++^*p* < 0.001. The results were statistically analyzed using the one-way analysis of variance (ANOVA) (biological replicates *n* = 3) followed by Tukey's multiple comparison test using GraphPad Prism v.9. Significant difference observed between *L. rhamnosus* GG and control (Pritenase K) is indicated: ****p < 0.0001.

Genomic analysis data show genes encoding protease activity in Renuspore^®^ include *clpC, clpP, clpX*, and *prp*. The assay determining the protease activity is based on the principle that the measured increase in fluorescence at Ex/ Em 485/535 nm is proportional to the proteinase activity. Renuspore^®^ showed a significant amount of extracellular protease activity when compared with *L. rhamnosus* GG [Figure 7, *F*_(2, 6)_ = 108.1, *p* < 0.0001].

### 3.9. Enzymatic profiling

Renuspore^®^ showed a range of peptidase, esterase, phosphatase, proteinase, lipase, and glycosidase activities (Table 2). Renuspore^®^ was characterized by strong esterase, α-chymotrypsin, alkaline phosphatase, phosphohydrolase, α-galactosidase, and β-galactosidase activities. From *in silico* analysis, genes encoding β-galactosidase, α-galactosidase, esterase, and peptidase were also identified although this did not directly correlate with the *in vitro* test results.

### 3.10. Free amino acids (FAAs) and short-chain fatty acids (SCFAs)

A metabolomic analysis revealed the proteolytic capability of Renuspore^®^ toward milk proteins generating a diverse range of amino acids. Out of 44 compounds, 30 were found to be statistically significant in Renuspore^®^ (Table 4, *p* < 0.05).

**Table 4:** Renuspore^®^ increased FAA in UHT fermented milk compared with the control (uninoculated UHT milk): ^*^p ≤ 0.05.

**Compound**	***p*** **-value**	***t*** **_(df)_ = *t* ratio**
Alanine	0.0142	*t*_(4)_ = 4.158
glycine	0.2862	*t*_(4)_ = 1.230
Valine	0.0000	*t*_(4)_ = 22.01
Leucine	0.0002	*t*_(4)_ = 12.84
Isoleucine	0.0001	*t*_(4)_ = 16.65
Threonine	0.0105	*t*_(4)_ = 4.545
Proline	0.0096	*t*_(4)_ = 4.658
Aspartic acid	0.0153	*t*_(4)_ = 4.067
Glutamic acid	0.0170	*t*_(3)_ = 4.817
Methionine	0.0009	*t*_(4)_ = 8.979
Phenylalanine	0.0027	*t*_(4)_ = 6.622
Lysine	0.0002	*t*_(3)_ = 21.34
Histidine	0.0295	*t*_(3)_ = 3.922
Tyrosine	0.0005	*t*_(4)_ = 10.43
Tryptophan	0.0012	*t*_(4)_ = 8.126
Cystine	0.0132	*t*_(4)_ = 4.243
Ornithine	< 0.000001	*t*_(3)_ = 153.0
Serine	0.0024	*t*_(4)_ = 6.871
Fumaric acid	0.0173	*t*_(4)_ = 3.917
4-Aminobenzoic acid	0.1161	*t*_(4)_ = 2.000
Succinic acid	0.0065	*t*_(4)_ = 5.205
Lactic acid	>0.9999	
2-Oxoglutaric acid	0.0003	*t*_(3)_ = 19.67
2-Phosphoenolpyruvic acid	0.0048	*t*_(3)_ = 7.584
Malic acid	0.0198	*t*_(4)_ = 3.760
cis-Aconitic acid	0.0002	*t*_(4)_ = 13.73
Citric acid	0.0000	*t*_(4)_ = 45.11
Isocitric acid	0.0001	*t*_(4)_ = 15.05
Alanyl-proline	0.0023	*t*_(3)_ = 9.774
Glycyl-proline	0.0008	*t*_(3)_ = 14.06
Octanoic acid	0.0647	*t*_(4)_ = 2.529
5-Aminovaleric acid	0.0000	*t*_(4)_ = 21.78
Benzoic acid	0.0000	*t*_(4)_ = 23.73
Phenylacetic acid	0.0005	*t*_(3)_ = 15.85
Decanoic acid	0.2097	*t*_(4)_ = 1.493
Decenoic acid	0.0211	*t*_(4)_ = 3.684
N-Acetyl-valine	0.0005	*t*_(4)_ = 10.13
Dodecanoic acid	0.1342	*t*_(4)_ = 1.874
Tetradecanoic acid	0.3271	*t*_(4)_ = 1.115
3-(methylthio)propionic acid	0.0001	*t*_(3)_ = 25.67
Pyruvic acid	0.0014	*t*_(3)_ = 11.54
4-methyl-2-oxopentanoic acid	0.0111	*t*_(3)_ = 5.623
3-Methyl-oxirane-2-carboxylic acid	0.0078	*t*_(3)_ = 6.368
4-Hydroxyphenylacetic acid	0.0014	*t*_(3)_ = 11.42

The results were statistically analyzed using multiple t-tests using the unpaired parametric, two-stage step—up (Benjamini, Krieger, and Yekutieli) (biological replicates n =3) using GraphPad Prism v.9.

A genomic analysis revealed the presence of oligopeptide (*Opp)* and dipeptide (*DtpP*) transporters, which are involved in the proteolysis of peptides to FAA by the action of peptidases. Aminopeptidases and metallopeptidases which are responsible for generating methionine and leucine or their derivatives were identified in the Renuspore^®^ genome. Proline peptidase (*pepP and pepX*), endopeptidase (*pepE and pepF*), and dipeptidase (*pepV and pepD*) were also found in the Renuspore^®^ genome.

For the SCFA analysis, a total of nine compounds were identified, of which only three were found to be significantly increased by Renuspore^®^ (Table 5). Interestingly, these were acetic acid, propionate, and 2-methyl-propionate. Propionate is usually associated with amino acid metabolism, specifically alanine and valine (Tavaria et al., 2002). A few esterases and lipases potentially related to the metabolic pathways of these SCFAs were identified in Renuspore^®^'s genome. Esterase A (*estA*), which shows relatively high activity toward β-naphthyl butyrate, and tannase/feruloyl esterase, which hydrolyzes bonds of tannic acid, feruloyl-arabinose ester bond in arabinoxylans, as well as the feruloyl–galactose and feruloyl–arabinose ester bonds in pectin, were identified. Lipase (*tesA*) belongs to class 3, which has been reported to hydrolyze long-chain acyl-triglycerides into di- and monoglycerides, glycerol, and free fatty acids (Cadwallader and Singh, 2009).

**Table 5:** SCFA increased in Renuspore^®^ UHT-fermented milk samples: ^*^p ≤ 0.05.

**Compound**	***p*** **-value**	***t*** **_(df)_ = *t* ratio**
Acetic acid	0.0114	*t*_(4)_ = 4.433
Formic acid	0.2552	*t*_(4)_ = 1.327
Propionate	0.0003	*t*_(4)_ = 12.23
2-methyl- propionate	0.0001	*t*_(4)_ = 17.23
Butyrate	0.3387	*t*_(4)_ = 1.086
3-methyl-butyrate	0.0791	*t*_(4)_ = 2.344
4-methyl-pentanoic acid	0.7519	*t*_(4)_ = 0.3386
Hexanoic acid	0.7670	*t*_(4)_ = 0.3171
Heptanoic acid	0.3340	*t*_(4)_ = 1.098

The results were statistically analyzed using multiple t-test using unpaired parametric, two-stage step—up (Benjamini, Krieger, and Yekutieli) (biological replicates n = 3) using GraphPad Prism v.9.

## 4. Discussion

In this study, the general probiotic properties of Renuspore^®^ were characterized. A common property shared by many probiotic strains is the capacity to inhibit the growth of pathogens and positively impact the gut microbial composition by producing antimicrobial agents or metabolic compounds that suppress the growth of other microorganisms (O'Shea et al., 2012). Renuspore^®^ had antimicrobial activity against both Gram-negative and Gram-positive bacteria, with the capacity to control zoonotic, opportunistic intestinal, skin, and urinary tract pathogens such as *Salmonella, E. coli, S. aureus*, and *C. jejuni*. Probiotic secondary metabolites can act as strong antimicrobial agents against pathogens and can also indirectly boost the host's defenses by modulating the host's immune or epithelial cells (Machado et al., 2021). Although classic antimicrobial compounds such as bacteriocins or lantibiotics produced by *Bacillus* species (Caulier et al., 2019) were not detected in Renuspore's^®^ genome, *in vitro* results suggest that it can inhibit the growth of several pathogens. Recent studies demonstrated how propionate and acetic acid had bactericidal activity against MRSA, *E. coli, Salmonella typhimurium*, and *C. albicans* independently of pH changes (Wali and Abed, 2019; Langfeld et al., 2021). Therefore, it is possible that high levels of acetic acid and propionate produced by Renuspore^®^ could be the reason for the antimicrobial activity observed in this study. It is essential for a probiotic in human nutrition to modulate metabolite production (Markowiak-Kope and Sli, 2020). An *In vitro* study made in UHT milk demonstrated significant levels of propionate production (Table 5). Consequently, Renuspore^®^ ingestion may increase propionate production in the gut and promote its anti-inflammatory properties and help lower lipogenesis and cholesterol levels in serum (Hosseini et al., 2011). Propionate production was likely linked to Renuspore^®^ esterolytic activity, with genes encoding esterase A and lipases.

Renuspore^®^ also demonstrated strong proteolytic activity against proteins present in milk, generating 30 free amino acids and carboxylic acids. The 30 free amino acid compounds increased in this *in vitro* test are a direct result of protease and peptidase activities. With its high proteolytic activity, Renuspore^®^'s presence in the gut could increase levels of FAA, which in turn would help regulate digestion, support the immune system, enhance mucous integrity and function, or act as an energy source for the human body (Kim et al., 2019). The phenotypic findings involved in the metabolism and transport of carbohydrates, proteins, and lipids do not always affiliate with specific genes. The *in vitro* studies in Renuspore^®^ did not completely infer the presence and absence of the associated genes. The disparity between the genome and *in vitro* analysis could be due to gene variations and additional protein-dependent sugar uptake systems with overlapping substrate or host conditions adaptability (Kilic et al., 2007; Buron-Moles et al., 2019). There are genes encoding enzymes identified as involved in trehalose, lactose, and inositol metabolism, whereas our investigation using API 50 CH indicated Renuspore^®^'s weak ability to ferment trehalose with no fermentation achieved for lactose or inositol. The reason for this disagreement could be that the set of genes involved in the uptake and metabolism of these sugars are incomplete, or these were false negatives and should be confirmed with a conventional phenol red broth or other more sensitive methods. In contrast, with other properties of many probiotics, Renuspore^®^ vegetative cells were unable to adhere to the intestinal cells or their secreted mucous, although they showed moderate adherence in their spore form. Therefore, in the intestinal tract, the spore form could adhere and germinate to vegetative cells, which would offer their potential probiotic effects. Since the vegetative form has a low adherence capacity, it is possible that Renuspore^®^ would be able to sequester heavy metals from the gut and then be washed out, helping remove these elements from the intestine.

In addition, beneficial bacterial metabolites and enzymes can influence the metabolism of xenobiotics and reactive oxygen species directly or indirectly by regulating the expression and function of key antioxidant liver enzymes (Clarke et al., 2019). Environmental contaminants such as heavy metals, nitrite, ammonia, and pesticides enter the food chain and can cause oxidative stress to the body. Oxidative stress then leads to cell death, and ingestion of these toxic heavy metals can cause multiorgan failure and immune system suppression (Syed and Chinthala, 2015). Probiotics have been known to offer protection against reactive oxygen species and oxidative stress and to be able to scavenge these molecules. *Lactobacillus* species have been reported to have antioxidant properties that work against heavy metal-induced oxidative stress in mice (Srednicka et al., 2021). Renuspore^®^ has been shown to have high levels of antioxidant molecules and glutathione compared with *L. rhamnosus* GG (Figure 1). These results correlated with the presence of antioxidant enzymes encoded in the Renuspore^®^ genome, suggesting that this probiotic strain could offer oxidative stress protection in the gut, shield cellular macromolecules from reactive oxygen and nitrogen species, and directly neutralize oxidative chemicals (Demirkol and Ercal, 2014). All these findings on Renuspore^®^ could possibly prevent leaky gut, intestinal disorders, cell damage, and diseases caused by reactive oxidative species imbalance in the body.

Since pollutants such as heavy metals are ubiquitous and unavoidable, various strategies including microbial bioremediation are being researched and implemented for the treatment and prevention of toxin exposures (Bisanz et al., 2014). Probiotic detoxification mechanisms include either sequestration to the bacterial cell wall or biosorption of metal ions into the bacterial cell wall which is followed by bioaccumulation within the bacterial cell through cell membrane transition (Abdel-Megeed, 2021). Several studies reported that *Lactobacillus* and *Saccharomyces* species have the potential to eliminate heavy metals either by binding or by biosorption (Bhakta et al., 2012; Alcántara et al., 2017; Duan et al., 2020). Similarly, *in vitro* studies showed the capacity of Renuspore^®^ to remove lead and mercury from the environment (Figure 3). This capacity to remove non-essential heavy metals could be related to the presence of *mer* and *ars* operons in the Renuspore^®^ genome (Giovanella et al., 2016), which encode proteins involved in mercury or lead regulation, binding, or degradation (Boyd and Barkay, 2012). Moreover, since Gram-positive bacteria have higher metal-binding activity, the affinity mechanism in Renuspore^®^ can act as a physical barrier in the gut to modify the absorption and metabolism of heavy metals (Abdel-Megeed, 2021). In addition, higher levels of glutathione production by Renuspore^®^ can support the transportation of mercury out of cells, facilitates the excretion of organic and toxic metal pollutants from the body, and can directly neutralize oxidative chemicals (Demirkol and Ercal, 2014).

Considering the xenobiotic contamination in the food chain, ammonia and nitrites are commonly present in the waste from poultry and aquaculture which can pollute soil and water ecosystem including the shrimps and fishes that humans consume (Anwar et al., 2021). In addition, ammonia and nitrites present in meat and cheese in the form of additives and flavoring can be toxic to human health (Karwowska and Kononiuk, 2020). Most bacterial species rely on xenobiotics for energy sources, and they have particular genes, enzymes, and degradative mechanisms to partially or completely metabolize xenobiotics. *B. subtilis* species were used to maintain (Zokaeifar et al., 2014) and decrease the concentrations of ammonia, nitrite, and nitrate levels in laying hens (Mi et al., 2019). The presence of a gene encoding the enzyme nitrite reductase, *NirD*, could explain how Renuspore^®^ was able to reduce and degrade nitrite (Hou et al., 2013) from the environment. Finally, microbial degradation has proven to be effective at degrading contaminants from industrial effluents such as chloronitrophenol (Arora, 2012). Data from the 4-Chloro-2-nitrophenol (4C2NP) decolorization result show the degradation capacity of Renuspore^®^ with respect to time (Figure 6). A study from the genomics and transcriptomics on *Rhodococcus* species suggests that 4-nitrophenol breaks down into acetyl-coA and succinate by nitrocatechol through *Nph*-specific genes (Miglani et al., 2022), facilitating the further metabolism of this hazardous pesticide. All these endocrine disruptors that are present in the environmental chemicals may contribute to the ecosocionomic burden by increasing human diseases and disabilities (Kassotis et al., 2020). Consumption of the probiotic strain Renuspore^®^ could potentially facilitate effective detoxification of such undesirable metabolites and reduced accumulation of environmental contaminants, thus reducing biological and physiological complications.

## 5. Conclusion

Globally, there is an unmet need for products to help cleanse and detoxify environmental contaminants in the food and water supply chains. Applications for microbes in the bioremediation of environmental contaminants represent a viable solution. More specifically, on an individual level, the role of probiotics in the removal of unwanted intrinsic and extrinsic toxins from the body is a very desirable trait. Renuspore^®^ may act as a catalyst in enhancing the natural biodegradation or sequestration of xenobiotics from our gut, and/or chelating and removing heavy metals by ion exchange, adsorption, or metal-binding proteins. Renuspore^®^ can also break down the desired pollutant through various enzymatic pathways and biochemical reactions. This xenobiotic bioreduction capacity of Renuspore^®^ represents natural technology to protect our health from contaminants present in the food chain. The *in silico* and *in vitro* data presented herein demonstrate the antimicrobial, antioxidant, and bioremediation properties of Renuspore^®^. Further beneficial probiotic effects include strong enzymatic activity against proteins, lipids, and carbohydrates to assist in food digestion, and the generation of various biomolecules known to be important in nutrient absorption. In summary, these findings suggest that Renuspore^®^ represents a strong probiotic candidate for facilitating gut and overall body health.

## Data availability statement

Publicly available datasets were analyzed in this study. This data can be found here: NCBI GenBank (NZ_JABBNK010000001) https://www.ncbi.nlm.nih.gov/nuccore/NZ_JABBNK010000001.

## Author contributions

AS, SM, EK, and JC performed the sample analysis. JD and KR were the advisor for the study and reviewed the manuscript. AS contributed to the writing of the manuscript. All authors contributed to the article and approved the submitted version.
